# Immunity to Non-Dengue Flaviviruses Impacts Dengue Virus Immunoglobulin G Enzyme-Linked Immunosorbent Assay Specificity in Cambodia

**DOI:** 10.1093/infdis/jiae422

**Published:** 2024-09-19

**Authors:** Camila D Odio, Christina Yek, Chloe M Hasund, Somnang Man, Piseth Ly, Sreynik Nhek, Sophana Chea, Chanthap Lon, Charlie Voirin, Rekol Huy, Rithea Leang, Chea Huch, Elaine W Lamirande, Stephen S Whitehead, Fabiano Oliveira, Jessica E Manning, Leah C Katzelnick

**Affiliations:** Viral Epidemiology and Immunity Unit, Laboratory of Infectious Diseases, National Institute of Allergy and Infectious Diseases, National Institutes of Health, Bethesda, Maryland, USA; International Center of Excellence in Research, National Institute of Allergy and Infectious Diseases, National Institutes of Health, Phnom Penh, Cambodia; Laboratory of Malaria and Vector Research, National Institute of Allergy and Infectious Diseases, National Institutes of Health, Bethesda, Maryland, USA; Viral Epidemiology and Immunity Unit, Laboratory of Infectious Diseases, National Institute of Allergy and Infectious Diseases, National Institutes of Health, Bethesda, Maryland, USA; International Center of Excellence in Research, National Institute of Allergy and Infectious Diseases, National Institutes of Health, Phnom Penh, Cambodia; International Center of Excellence in Research, National Institute of Allergy and Infectious Diseases, National Institutes of Health, Phnom Penh, Cambodia; International Center of Excellence in Research, National Institute of Allergy and Infectious Diseases, National Institutes of Health, Phnom Penh, Cambodia; International Center of Excellence in Research, National Institute of Allergy and Infectious Diseases, National Institutes of Health, Phnom Penh, Cambodia; International Center of Excellence in Research, National Institute of Allergy and Infectious Diseases, National Institutes of Health, Phnom Penh, Cambodia; Viral Epidemiology and Immunity Unit, Laboratory of Infectious Diseases, National Institute of Allergy and Infectious Diseases, National Institutes of Health, Bethesda, Maryland, USA; National Center for Parasitology, Entomology, and Malaria Control, Ministry of Health, Phnom Penh, Cambodia; National Center for Parasitology, Entomology, and Malaria Control, Ministry of Health, Phnom Penh, Cambodia; National Center for Parasitology, Entomology, and Malaria Control, Ministry of Health, Phnom Penh, Cambodia; Laboratory of Viral Diseases, National Institute of Allergy and Infectious Diseases, National Institutes of Health, Bethesda, Maryland, USA; Laboratory of Viral Diseases, National Institute of Allergy and Infectious Diseases, National Institutes of Health, Bethesda, Maryland, USA; International Center of Excellence in Research, National Institute of Allergy and Infectious Diseases, National Institutes of Health, Phnom Penh, Cambodia; Laboratory of Malaria and Vector Research, National Institute of Allergy and Infectious Diseases, National Institutes of Health, Bethesda, Maryland, USA; International Center of Excellence in Research, National Institute of Allergy and Infectious Diseases, National Institutes of Health, Phnom Penh, Cambodia; Laboratory of Malaria and Vector Research, National Institute of Allergy and Infectious Diseases, National Institutes of Health, Bethesda, Maryland, USA; Viral Epidemiology and Immunity Unit, Laboratory of Infectious Diseases, National Institute of Allergy and Infectious Diseases, National Institutes of Health, Bethesda, Maryland, USA

**Keywords:** dengue virus, West Nile virus, seroprevalence, cross-reactivity, specificity

## Abstract

**Background:**

Seroprevalence studies are the standard for disease surveillance, and serology determined eligibility for the first dengue vaccine. Expanding flavivirus co-circulation and vaccination complicate testing. We evaluate the accuracy of a common dengue virus serological assay, examine immunity to non-dengue flaviviruses as a contributor to decreased performance, and assess whether alternative cut points may improve assay performance.

**Methods:**

Children (n = 770) aged 2–9 years in Kampong Speu, Cambodia were enrolled in a prospective longitudinal study, and PanBio indirect dengue virus immunoglobulin G (IgG) enzyme-linked immunosorbent assay (ELISA) was performed. Plaque reduction neutralization tests (PRNTs) using dengue viruses were performed on a subset to assess the accuracy of the IgG ELISA, and PRNTs with Zika, Japanese encephalitis, and West Nile viruses evaluated immunity to non-dengue flaviviruses. Receiver operating curve analysis identified an alternative cut point to improve IgG ELISA accuracy.

**Results:**

The dengue IgG ELISA had a lower specificity than previously reported (58% vs 93%–100%). Of those with false-positive IgG results, 46% had detectable neutralizing antibodies against other flaviviruses including 14% against West Nile virus. A higher IgG cut point improved the test accuracy in this population.

**Conclusions:**

Physicians and public health authorities should be alert for West Nile in Cambodia. Immunity to non-dengue flaviviruses can impact dengue surveillance.

**Clinical Trials Registration:**

NCT03534245.

## Teaser

In this cohort of 770 Cambodian children aged 2–9 years, a dengue virus IgG ELISA had a lower specificity than previously reported (58% vs 93%–100%). Neutralizing antibodies against other flaviviruses contributed to this decreased accuracy and should be considered in dengue surveillance.

The genus *Orthoflavivirus* includes multiple pathogenic mosquito-borne viruses such as dengue viruses 1–4 (DENV1–4), Japanese encephalitis virus (JEV), West Nile virus (WNV), Zika virus (ZIKV), and yellow fever virus (YFV) [1]. With expanding vector habitats, known flaviviruses are rising in global incidence and novel flaviviruses are emerging [2, 3]. These flaviviruses commonly co-circulate, and the antibodies induced by one exposure may cross-react with others in the genus [4]. Additionally, affected areas use vaccines to protect against JEV and YFV, further complicating serology. As flaviviruses expand their range and vaccination increases, differentiating true exposure from cross-reactivity is not only difficult, but also increasingly important to guide diagnostic, preventative, and therapeutic measures.

Accurately characterizing population-level immunity to DENV is important for current dengue vaccination efforts. Dengvaxia (Sanofi Pasteur), the first widely approved dengue vaccine, was originally recommended in 2016 by the World Health Organization (WHO) Strategic Advisory Group of Experts panel for use in areas with ≥70% DENV seroprevalence in children aged 9 and older, with seroprevalence most often measured using common immunoglobulin G (IgG) enzyme-linked immunosorbent assay (ELISAs) [5]. When it was later shown that Dengvaxia increases the risk of severe disease in DENV-naive individuals, the WHO recommended the use of highly specific individual-level testing of DENV immunity to confirm vaccine eligibility, along with use in high-risk populations in endemic areas [6, 7]. Notably, Sanofi Pasteur has recently decided to stop producing Dengvaxia [8]. A second DENV vaccine, QDENGA (Takeda), has been prequalified by the WHO with recommended use in children aged 6 to 16 years in areas with high endemicity and transmission intensity [9]. Given the lack of observed vaccine-induced protection against DENV3 in seronegative individuals and the unknown protection against DENV4 [10], a strategy based on population-level estimates of endemicity may not be the safest choice for seronegative individuals. Regardless of whether individual measures or population-level estimates of dengue immunity are used, accurate serological testing is critical to determine vaccine appropriateness.

Population-level serosurveys for DENV conducted for surveillance purposes generally use commercial IgG ELISAs. The plaque reduction neutralization test (PRNT), which measures neutralizing antibodies (nAbs) to DENV, is considered the gold standard for evaluating specificity but requires intensive and specialized labor. The PanBio indirect DENV IgG ELISA (Abbott) is one of the most commonly used assays for measuring DENV immunity, and the manufacturer reports 100% specificity based on 108 DENV-naive individuals from endemic areas [11]. Separate work demonstrated 99% specificity of this ELISA using DENV1–4 PRNT_50_ ≥ 10 as the standard for DENV immunity in a cohort of 534 individuals from both the United States (n = 229 individuals aged 18–45 years) and dengue-endemic regions (n = 106 individuals aged 2–14 years and n = 199 individuals aged 9–16 years) before 2016 [12]. However, the PanBio indirect DENV IgG ELISA yields higher false-positivity rates when evaluated in individuals with immunity to other flaviviruses, including those who had received an inactivated JEV vaccine (3%), or had immunity against ZIKV (34%) or WNV (51%) [12]. The degree to which immunity to other flaviviruses affects DENV serosurveys is dependent on the site, and a major challenge is that the extent of circulation of other flaviviruses is often unknown. For instance, an observational study of children aged 9–14 years in the Philippines in 2017 used a PRNT_70_ ≥ 40 as the indicator of DENV immunity and reported a lower ELISA specificity of 93.4% [13]. Of the false-positive samples, 64% had nAb against ZIKV or JEV, in a region where ZIKV was not thought to be widespread. Thus, although the PanBio ELISA has a reportedly high specificity, this number may vary with flavivirus cross-reactivity and expanding co-circulation or vaccination.

Here, we leverage a cohort of young children in Cambodia to evaluate the accuracy of a commonly used serological assay, examine immunity to non-dengue flaviviruses as a potential contributor to decreased performance, and assess whether an alternative cut point may improve the assay's performance. Specifically, Cambodia is highly dengue endemic, ZIKV was recently found to co-circulate, and JEV is endemic. JEV vaccination campaigns with a live-attenuated JEV vaccine, SA14-14-2, started around 2014, and WNV nAbs have been identified in birds but not humans [14–17]. Thus, this work informs important considerations for assessing dengue immunity in areas with exposure to multiple flaviviruses.

## METHODS

The study protocol was approved by the institutional review boards at the US National Institutes of Health and the National Ethics Committee on Human Research in Cambodia. The guardians of all pediatric participants provided signed informed consent to participate in the study.

Between July and August of 2018, 771 children aged 2–9 years living in Kampong Speu, Cambodia were enrolled in a prospective longitudinal cohort (NCT03534245) [18]. At entry, the PanBio indirect DENV IgG ELISA was performed on the 770 individuals with sera available. This assay was performed using an initial serum dilution of 1:100 and following the manufacturer’s instructions. The ELISA index values were determined by dividing the sample absorbance by the cutoff value and multiplying by 10. The cutoff value is the average absorbance of the triplicates of the calibrator multiplied by the calibration factor.

ELISA IgG index values were then used to categorize participants per the manufacturer's instruction: ELISA > 1.1, DENV IgG positive; ELISA 0.9–1.1, equivocal; and ELISA < 0.9, negative. Of the 770 individuals tested, n = 273 had an ELISA > 1.1, n = 13 had an ELISA 0.9–1.1, n = 44 had an ELISA 0.2–0.9, and n = 440 had an ELISA < 0.2 (Table 1). DENV1–4 PRNTs were performed on all individuals with ELISA > 0.9 (n = 286) and on a subset of individuals with ELISA < 0.9 (n = 25 with ELISA 0.2–0.9 and n = 25 with ELISA < 0.2). In our cohort, 100% of the 25 individuals tested with ELISA < 0.2 had DENV PRNT_50_ < 10, which is consistent with previous work showing that 99% of those with ELISA < 0.2 had DENV PRNT_70_ < 40 [13]. Given this validation, all individuals with ELISA < 0.2 were considered DENV1–4 naive.

**Table 1.** Number of Individuals With DENV IgG ELISA and PRNT Testing, and Performance of the ELISA Test in Determining Serostatus Compared to the Gold Standard PRNT With and Without ELISA Equivocal Samples (ELISA Values 0.9–1.1)

				All Samples		Excluding Equivocal	
ELISA Result	ELISA Value	ELISA, n	PRNT, n	PRNT<10 (n = 67)	PRNT≥10 (n = 269)	PRNT<10 (n = 63)	PRNT≥10 (n = 260)
Negative (DENV 1-4 naive)	<0.2	440	25	39 True negative	24 False negative	35 True negative	15 False negative
Negative	0.2–0.9	44	25				
Equivocal	0.9–1.1	13	13				
Positive	>1.1	273	273	28 False positive	245 True positive	28 False positive	245 True positive
				Specificity: 58% (46%–70%)	Sensitivity: 91% (87%–94%)	Specificity: 56% (42%–68%)	Sensitivity: 94% (91%–97%)

PRNT ≥ 10 indicates that the individual had this titer against ≥1 DENV serotype.

Abbreviations: DENV, dengue virus; ELISA, enzyme-linked immunosorbent assay; PRNT, plaque reduction neutralization test.

PRNTs were performed using the following viruses grown in Vero cells: DENV1 West Pac prototype strain (recombinant), Genbank AY145121; DENV2 New Guinea C prototype strain, Genbank AF038403; DENV3 Sleman/1978 (recombinant) with Vero adapted mutation at position 7164, Genbank KT452798; DENV4 Dominica/1981, Genbank AF326573; ZIKV-Paraiba/2015, Genbank AF326573; WNV/DEN4Δ30 (E and prM proteins of WNV from Genbank AF196835 in a DENV4 backbone of Genbank AF326827) [19]; WNV NY99, Genbank AF196835; and the JEV vaccine strain SA14-14-2, Genbank MH258849 [20]. Antigenic cartography analyses have shown that these DENV prototype strains are overall representative of circulating strains [21]. Nevertheless, laboratory-adapted prototype strains can be more sensitive to neutralization than circulating strains. Due to biosafety limitations, a chimeric WNV vaccine strain was used to screen individuals for WNV nAbs [22]. Sera that neutralized the WNV chimeric strain (n = 14) were then tested against the wild-type WNV strain to confirm immunity (Supplementary Table 1). Only sera that neutralized the wild-type WNV strain were considered immune to WNV (n = 7).

The PRNT was performed as described previously [23, 24]. Briefly, 24 hours prior to the experiment, Vero cells were plated at 1.7×10^4^ per well and incubated at 37°C. Sera were heat inactivated at 56°C for 30 minutes. Sera were then 4-fold serially diluted for ZIKV and DENV1–4 assays (1:10 to 1:640 final dilution) and JEV and WNV (1:10 to 1:10 240 final dilution), and then added to the wells, starting at a dilution of 1:5, followed by the addition of an equal volume of virus. Thus, the lower limit of detection was a dilution of 1:10. Sera-virus mixtures were incubated for one hour at 37°C, then added to confluent cells and incubated for one hour. Due to differences in laboratory protocols, nAbs to the wild-type WNV were tested on Vero cells plated 48 hours prior to the experiment, and sera admixtures were incubated on cells for 30 minutes. Following infection, 1% methycellulose overlay was added to each well, and plates were incubated for two days for DENV1–4, 3–4 days for ZIKV, 40 hours for JEV, 50 hours for the chimeric WNV, and 24 hours for the wild-type WNV. Cells were then fixed in 80% methanol and blocked with 5% nonfat dried milk. Next, cells were immunostained using mouse panflavivirus monoclonal antibodies 4G2 and 2H2, secondary horseradish peroxidase (HRP)-labelled goat anti-mouse IgG antibody (Jackson ImmunoResearch Laboratories), and developed using TrueBlue HRP substrate. Each well image was collected using a Cellular Technology Limited machine, and the Viridot plaque counter package in R was used for automated plaque counting [25]. The drc package in R was then used to estimate the PRNT_50_ titers by 4-parameter logistic regression. For quality control, JEV and WNV neutralization curves with an *R*^2^ > 0.75 and a Hill slope with an absolute value of more than 0.5 were included in subsequent analyses. Samples that did not meet these criteria were repeated. nAb titers were defined as the reciprocal of the calculated dilution wherein virus infectivity was reduced by 50% (PRNT_50_). PRNT_50_≥1:10 (reported as PRNT_50_≥10) against any DENV serotype was considered immune to that serotype. Seropositivity to any non-dengue flavivirus was defined as PRNT_50_≥10, which is consistent with prior classifications [26–28]. To assess individuals with PRNT_50_≥10 against multiple flaviviruses, PRNT_90_ values were calculated. For JEV and WNV, PRNT_90_ values were calculated by plotting a 4-parameter logistic regression curve in PRISM (version 9 for macOS) from the 6 dilution points. The concentration at which 90% of virus was neutralized was assessed and recorded. For DENV and ZIKV, PRNT_90_ values were calculated using an Excel-based titer calculator for a 2-point regression analysis.

All DENV and ZIKV assays were performed at the International Center of Excellence in Research in Phnom Penh, Cambodia using one human serum sample that was known positive for DENV1–4 and ZIKV and one human serum sample that was pan-negative on all experiments. The JEV and chimeric WNV assays were all performed at the Viral Epidemiology and Immunity Unit in Bethesda, MD, and the wild-type WNV assays were performed with support from the Laboratory of Viral Diseases in Bethesda, MD. Antibodies against JEV and chimeric WNV were measured in all individuals who had DENV1–4 titers < 10 and ELISA > 1.1 (false-positive group, n = 28), all individuals with DENV1–4 titers of 10 to 20 and ELISA > 1.1 (low DENV nAb, n = 21), and a random subset of individuals with ELISA < 0.2 (naive, n = 50). One sample of human sera known to be seronegative to all flaviviruses of interest was used as a negative control in all JEV and WNV experiments. One human serum sample from a known JEV vaccine recipient was used as the positive control for the JEV experiments, and a polyclonal rabbit sera specific to WNV was used as the positive control in the chimeric and wild-type WNV experiments.

The performance of the ELISA IgG compared to the DENV1–4 PRNT gold standard test was evaluated by calculating the sensitivity and specificity of the ELISA test. Here, the sensitivity is the proportion of people with DENV immunity by PRNT who test positive by ELISA, and the specificity is the proportion of people with no DENV immunity by PRNT who test negative by ELISA. Initially, the sensitivity and specificity were measured using the manufacturer's recommended cut point of ELISA > 1.1 as indicative of positive anti-DENV immunity. Next, these values were calculated after excluding the individuals with ELISA values between 0.9 and 1.1 because these were considered equivocal by the manufacturer.

Given the ELISA IgG is measured on a continuous scale, it is possible that a cut point other than 1.1 could improve the sensitivity and specificity of this test in our population. To assess the optimal ELISA IgG cut points for DENV positivity, receiver operating characteristic (ROC) curves were generated. Specifically, ROC curves plot the sensitivity versus 1 − specificity of each test [29]. From this plot, the area under the curve (AUC) provides an estimate of the accuracy of the test and can be compared among various tests. Higher AUCs indicate a more accurate test.

Tables with resulting *P* values were generated in RStudio for macOS (2022.07.1, build 554) using the tidyverse and gtsummary packages. Sensitivity and specificity results were generated using the epiR package, the optimal ELISA cut point and ROC curve were identified with the cutpointr package, using the maximize sensitivity and specificity method. Heatmaps were generated in PRISM (version 9 for macOS).

## RESULTS

We evaluated performance of the PanBio IgG ELISA in detecting DENV-specific immunity in a cohort of 770 children in Cambodia (Table 1). When using the PRNT as the reference test, the IgG ELISA had a sensitivity of 91% (95% confidence interval [CI], 87%–94%) and a specificity of 58% (95% CI, 46%–70%). Individuals with a false-positive ELISA IgG had a mean age and ELISA value that fell between those of the true-negative and true-positive groups (Table 2 and Supplementary Table 2 presents these data by age group). Among those who had ELISA and PRNT data assessed (n = 336), there were n = 20 reverse transcription polymerase chain reaction (RT-PCR)-confirmed cases of symptomatic dengue with no differences in frequency among groups.

**Table 2.** **Demographics and Immune Profiles of Individuals who had ELISA≤1.1 and DENV PRNT_50_ Against all Serotypes<10 (True Negative), ELISA≤1.1 and DENV PRNT_50_ Against at Least 1 Serotype**≥**10 (False Negative), ELISA>1.1 and DENV PRNT_50_ Against all Serotypes<10 (False Positive), and ELISA>1.1 and DENV PRNT_50_ Against Any Serotype**≥**10 (True Positive)**

Characteristic	True Negative, (n = 39)	False Negative, (n = 24)	False Positive, (n = 28)	True Positive, (n = 245)	*P* Value^a^
Age, y, mean (SD)	5.67 (2.33)	5.67 (2.12)	5.96 (2.15)	6.80 (2.20)	.002
Sex, n (%)					.5
Female	20 (51)	12 (50)	10 (36)	126 (51)	
Male	19 (49)	12 (50)	18 (64)	119 (49)	
ELISA IgG value, mean (SD)	0.32 (0.36)	0.66 (0.36)	2.00 (0.72)	2.92 (0.84)	<.001
Dengue case, n (%)					.5
No dengue	30 (91)	16 (84)	22 (92)	156 (93)	
Dengue	3 (9.1)	3 (16)	2 (8.3)	12 (7.1)	
Unknown	6	5	4	77	

Unknown indicates that the participant was lost to follow-up prior to the final study visit.

Abbreviations: DENV, dengue virus; ELISA, enzyme-linked immunosorbent assay; PRNT, plaque reduction neutralization test.

^a^One-way ANOVA; Pearson χ^2^ test; Fisher exact test.

**Figure 1.**
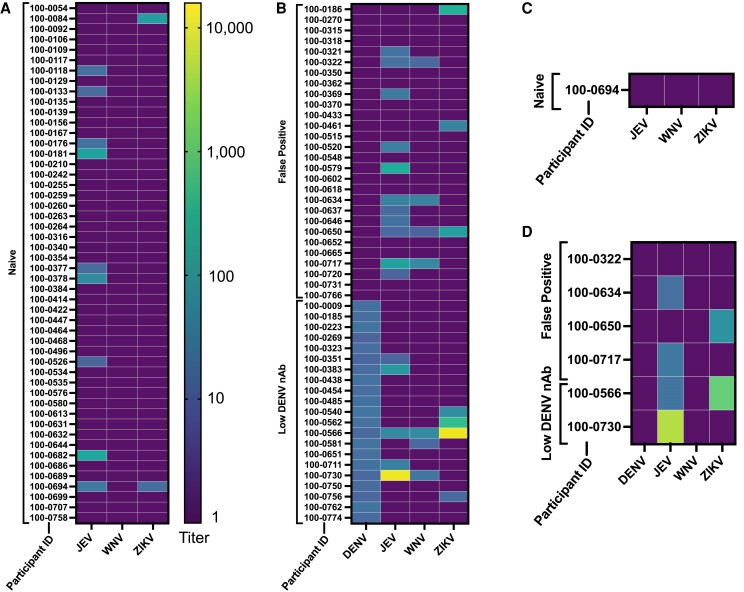
Neutralizing antibody titers against JEV, WNV, ZIKV, and DENV in individuals who were (*A* and *C*) DENV1–4 naive (ELISA < 0.2) or (*B* and *D*) false positive (ELISA >1.1, DENV PRNT_50_ against all serotypes < 10), or had low DENV nAb (ELISA >1.1, DENV PRNT_50_ 10–20 against ≥1 serotype) as measured by PRNT_50_ (*A* and *B*) and PRNT_90_ titers (*C* and *D*). WNV titers were measured using the chimeric strain for all individuals, and those with nAb against the WNV chimera were confirmed using the wild-type strain. Only positive WNV titers against the wild-type strain are reported. PRNT_90_ titers were only measured in those with nAb against ≥2 nondengue flaviviruses. Abbreviations: DENV, dengue virus; ELISA, enzyme-linked immunosorbent assay; JEV, Japanese encephalitis virus; nAb, neutralizing antibody; PRNT, plaque reduction neutralization test; WNV, West Nile virus; ZIKV, Zika virus.

We hypothesized that the ELISA false-positive results could be due to cross-reactivity against non-dengue flaviviruses. To test this, we compared the frequencies of nAbs against JEV, ZIKV, and WNV in the false-positive individuals versus n = 50 randomly chosen dengue-naive individuals with ELISA < 0.2 (Figure 1*A* and 1*B*). Consistent with prior classifications, seropositivity to non-dengue flaviviruses was defined as PRNT_50_≥10 [26–28]. Overall, 46% of the false-positive group had PRNT_50_≥10 against ≥1 non-dengue flavivirus versus 20% of the naive group (*P* = .020; Table 3). Although the false-positive group had higher percentages of individuals with positive JEV and ZIKV nAbs, only WNV nAbs were significantly more common than in the naive group (0% vs 14%, *P* = .014). To further assess these trends, we tested n = 21 individuals with DENV ELISA >1.1 and DENV PRNT_50_ between 10 and 20. This group has low DENV nAbs and has been considered DENV negative in other work [13, 18]. When compared to the false-positive group, the low DENV nAb group had similar frequencies of JEV, ZIKV, and WNV nAb (*P* ≥ .4 for all 3 nAbs; Supplementary Table 3 presents these data by age group).

**Table 3.** The Presence of Neutralizing Antibodies Against Non-Dengue Flaviviruses Compared in the Naive versus False-Positive Groups and Between the False-Positive and the Low DENV nAb Group (ELISA>1.1 and PRNT_50_ of 10–20 Against ≥1 Serotype)

	Naive (n = 50)	*P* Value^a^	False Positive (n = 28)	*P* Value^b^	Low DENV NAb (n = 21)
JEV nAb		.058		0.4	
Negative	41 (82)		17 (61)		16 (76)
Positive	9 (18)		11 (39)		5 (24)
ZIKV nAb		.3		0.4	
Negative	48 (96)		25 (89)		17 (81)
Positive	2 (4)		3 (11)		4 (19)
WNV nAb		**.014**		>0.9	
Negative	50 (100)		24 (86)		18 (86)
Positive	0 (0)		4 (14)		3 (14)
≥ 1 Nondengue flavivirus positive	10 (20)	**.020**	13 (46)	>0.9	9 (43)
≥ 2 Nondengue flavivirus positive	1 (2)	.053	4 (14)	0.7	2 (9.5)
JEV vaccine		.12		0.8	
Yes	2 (4)		3 (11)		3 (14)
No	2 (4)		2 (7)		2 (9.5)
Did not know	13 (26)		2 (7)		3 (14)
Unable to contact	33 (66)		21 (75)		13 (62)

Data are No. (%).

Abbreviations: DENV, dengue virus; JEV, Japanese encephalitis virus; nAb, neutralizing antibodies; WNV, West Nile virus; ZIKV, Zika virus.

^a^Fisher’s exact test comparing naive versus false-positive group.

^b^Fisher’s exact test comparing false-positive group versus low DENV nAb groups.

There were 7 individuals that had PRNT_50_≥10 against 2 or more non-dengue flaviviruses. To help elucidate the primary flavivirus exposure in these individuals, PRNT_90_ titers were calculated (Figure 1*C* and 1*D*) [4]. Of these, n = 2 had PRNT_90_<10 against all 4 flaviviruses, and n = 1 had PRNT_90_≥10 to multiple flaviviruses (JEV and ZIKV). Four individuals had PRNT_90_≥10 against only 1 flavivirus: n=1 to ZIKV and n=3 to JEV. There was also one individual with low DENV nAb and PRNT_50_≥10 against WNV only. Because this individual did not have PRNT_90_≥10 against either DENV or WNV, the primary infecting virus is unknown. Regardless of the WNV nAb source, this immunity was common and likely central to the decreased ELISA specificity observed.

Because immunity due to a vaccine may differ from that induced by natural infection, the false-positive, low DENV nAb, and n=50 naive individuals were evaluated for receipt of JEV vaccine (Table 3). However, due to difficulty recontacting the participants after study closure, only 4–5 individuals per group were able to provide data regarding JEV vaccination (Table 3). Thus, although the source of JEV immunity was unknown, we did find that immunity to other flaviviruses, particularly WNV, may contribute to high ELISA IgG values in individuals with undetectable and low DENV nAbs.

**Figure 2.**
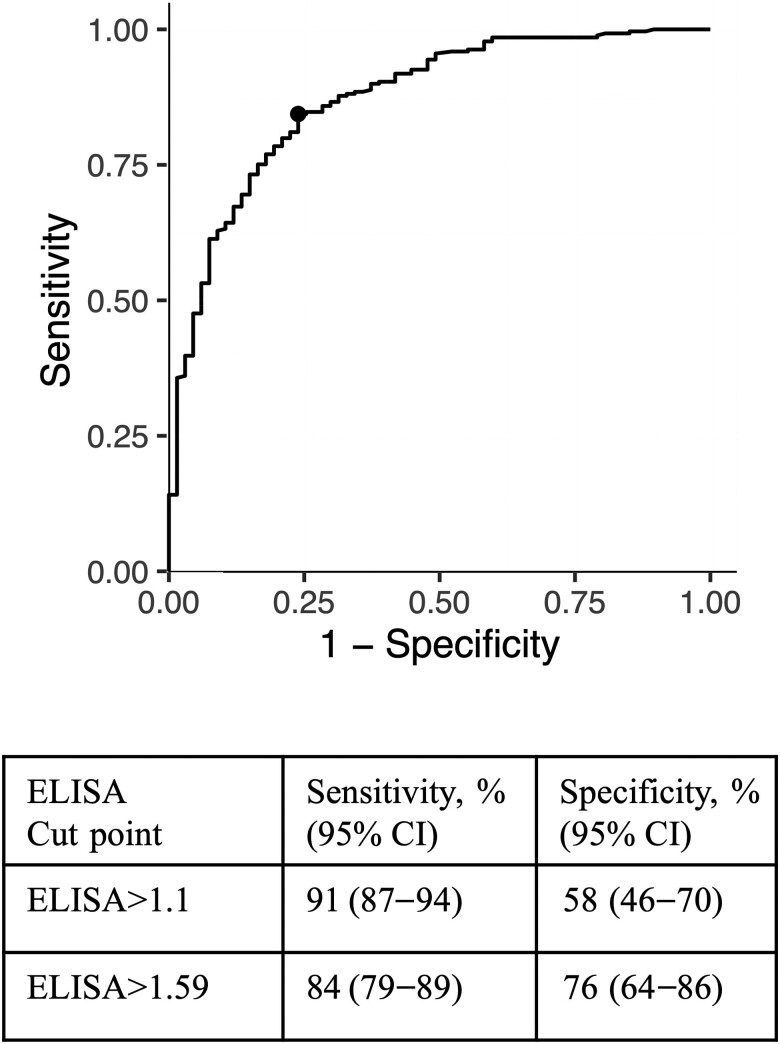
Receiver operating curve analysis for all individuals with ELISA and PRNT testing (n = 336). Sensitivity and specificity are reported for ELISA cut points of 1.1 versus 1.59. Abbreviations: CI, confidence interval; ELISA, enzyme-linked immunosorbent assay; PRNT, plaque reduction neutralization test.

Given the decreased specificity observed when using ELISA>1.1 as a cut point for DENV positivity, we generated a ROC curve that identified an alternative cut point of 1.59 for the ELISA (Figure 2). This higher ELISA cut point increased the specificity to 76%, but decreased the sensitivity to 84%. Thus, a higher cut point may be required when evaluating individuals with potential prior infection and/or vaccination with other flaviviruses.

## DISCUSSION

This unique study enrolled children aged 2–9 years living in a dengue-endemic region who were mostly DENV naive (68% of cohort), providing an opportunity to evaluate immunity measurements following natural infection and/or vaccination with other flaviviruses. In this cohort, we found that the PanBio DENV indirect IgG ELISA had a much lower specificity than reported in prior studies (58% vs 93%–100%) [12, 13]. This discrepancy is likely because one previous study primarily included individuals that were known to be flavivirus negative, and selecting samples this way may overestimate assay performance compared to a real-world setting [12]. Indeed, this previous study reported a 100% specificity of the ELISA. The second study was more representative of a local population, but the children were 9–14 years old and mostly DENV immune (89% immune) [13]. This high rate of DENV immunity compared to our cohort was likely related to their older age (9–14 years vs 2–9 years in the present work), giving them more time to be infected with DENV. Because they were highly DENV immune, DENV positivity by ELISA was less likely to be falsely positive, which resulted in a higher ELISA specificity of 94%. Thus, factors like age, flavivirus infection or vaccination, and DENV immunity within the population can cause variability in testing accuracy.

In our cohort, the high number of false-positive ELISAs was partially explained by the assay's detection of WNV nAb with potential contributions by ZIKV and JEV nAb. Notably, JEV vaccination can induce WNV cross-reactivity [30], and it is possible that the WNV nAb were induced by JEV vaccination or infection. However, the WNV nAbs could represent true WNV exposure, underlining the need for ongoing vigilance for WNV circulation in humans in Cambodia. Notably, half of the false-positive results remained unexplained, potentially due to waning immunity or infection by unidentified flaviviruses.

Given the high number of false-positive individuals, we performed a ROC curve analysis that recommended an ELISA cut point of 1.59. This cut point is much higher than the cut points of 1.1 used in the cohort with known flavivirus-naive individuals and 0.9 recommended by the study of mostly DENV-immune children [12, 13]. This finding indicates that cut points may vary with population immunity, and this must be considered when interpreting ELISA results.

Clinicians, investigators, and public health authorities should be aware that expanding flavivirus cocirculation and vaccination could increasingly impact serology results, especially in DENV-naive populations. Serosurveys conducted for vaccination campaigns to identify populations where DENV is endemic may overestimate dengue burden as a result of false positivity due to infection or vaccination with other flaviviruses. Such population-based strategies are of particular concern when identifying target populations for dengue vaccines where safety in DENV-seronegative individuals has not yet been confirmed. Adverse events in these individuals could greatly impact vaccine trust and uptake, as occurred with Dengvaxia [31].

For vaccines like Dengvaxia that are recommended for use only in DENV-seropositive individuals, pre-vaccination screening is required to determine vaccine eligibility, which allows individuals to make informed decisions about their own vaccine risk and benefit. In this cohort, even the higher ELISA cut point of 1.59 does not reach a specificity ≥90%, which is the minimum recommended by experts for prevaccine screening tests [32]. Instead, the ELISA IgG would need a cut point of 2.49 to reach a specificity of 90% (95% CI, 80%–96%). However, this cut point results in a sensitivity of 63% (95% CI, 57%–69%), which is much lower than minimum ≥90% sensitivity recommended by the experts for a pre-vaccine screening test. Thus, this PanBio ELISA IgG is not an appropriate pre-vaccine screening test, especially in populations with a high proportion of DENV-naive individuals and co-circulating flaviviruses. The safest strategy would likely be confirming previous exposure either by a virological assay or by two highly specific serological assays, such as the anti-DENV1–4 NS1 ELISA IgG and an IgG rapid test, as recommended by the US Centers for Disease Control and Prevention [7]. In summary, evaluation of false positivity due to infection with other emerging flaviviruses is critical to ensuring the safety of screening approaches.

Overall, our study demonstrates that the PanBio IgG ELISA and even PRNT results should be interpreted with caution in areas with flavivirus co-circulation and vaccines, and multiple tests may be required to confirm DENV seroprevalence.

## Supplementary Data

Supplementary materials are available at *The Journal of Infectious Diseases* online (http://jid.oxfordjournals.org/). Supplementary materials consist of data provided by the author that are published to benefit the reader. The posted materials are not copy edited. The contents of all supplementary data are the sole responsibility of the authors. Questions or messages regarding errors should be addressed to the author.

## Supplementary Material

jiae422_Supplementary_Data

## Notes

***Acknowledgments*.** The authors thank Patrick Mpingabo for his support establishing ELISA assays to evaluate samples for antibodies against the nonstructural-1 proteins of JEV, ZIKV, and WNV. These assays were not ultimately used in the article. We also thank Kelsey Lowman for her support completing the WNV and JEV PRNT assays.

***Disclaimer*.** Merck had no input on this article.

***Financial support*.** This work was supported by the Intramural Research Program at the National Institute of Allergy and Infectious Diseases (NIAID). Additionally, we submitted a proposal of our own creation to Merck ISS requesting funding to expand the neutralization data from our preexisting cohort study (funded by NIAID). Merck granted this award and transferred payment to NIAID. These funds were used to purchase supplies for neutralization assays and support ancillary operational staff to execute these assays.
