# Correction to: Immunity to Non-Dengue Flaviviruses Impacts Dengue Virus Immunoglobulin G Enzyme-Linked Immunosorbent Assay Specificity in Cambodia

**DOI:** 10.1093/infdis/jiae492

**Published:** 2024-10-15

This is a correction to: Camila D Odio *et al.*, Immunity to Non-Dengue Flaviviruses Impacts Dengue Virus Immunoglobulin G Enzyme-Linked Immunosorbent Assay Specificity in Cambodia, *The Journal of Infectious Diseases*, 2024; jiae422, https://doi.org/10.1093/infdis/jiae422

The author Fabiano Oliveira was originally listed as L. Fabiano Oliveira. This has been corrected.

