# Supplementary material for: Immunity to Non-Dengue Flaviviruses Impacts Dengue Virus Immunoglobulin G Enzyme-Linked Immunosorbent Assay Specificity in Cambodia
Source: J Infect Dis. 2024 Sep 19;231(2):e337–44. doi: 10.1093/infdis/jiae422 (PMC11841641; doi:10.1093/infdis/jiae422)
Supplement: jiae422_Supplementary_Data [file jiae422_supplementary_data.zip › TableS1.docx]

Supplemental Table 1. PRNT_50_ titer against the chimeric and wild-type West Nile virus (WNV) strains. Positive titers (PRNT_50_≥10) are in **bold-type** font.

| Sample ID | Chimeric WNV PRNT_50_ Titer | Wild-type WNV PRNT_50_ Titer |
| --- | --- | --- |
| 100-0009 | **30.38** | 1 |
| 100-0321 | **10.45** | 1 |
| 100-0322 | **55.96** | **11.8** |
| 100-0383 | **98.73** | 1 |
| 100-0562 | **241.65** | 1 |
| 100-0566 | **50.79** | **48.59** |
| 100-0581 | **14.59** | **12.12** |
| 100-0634 | **81.93** | **33.57** |
| 100-0637 | **13.7** | 1 |
| 100-0646 | **27.52** | 1 |
| 100-0650 | **17.56** | **10.0** |
| 100-0711 | **51.62** | 1 |
| 100-0717 | **25.67** | **40.21** |
| 100-0730 | **62.1** | **19.75** |
