# Supplementary material for: Immunity to Non-Dengue Flaviviruses Impacts Dengue Virus Immunoglobulin G Enzyme-Linked Immunosorbent Assay Specificity in Cambodia
Source: J Infect Dis. 2024 Sep 19;231(2):e337–44. doi: 10.1093/infdis/jiae422 (PMC11841641; doi:10.1093/infdis/jiae422)
Supplement: jiae422_Supplementary_Data [file jiae422_supplementary_data.zip › TableS2.docx]

Supplemental Table 2. Anti-DENV immune profiles and dengue cases by age in individuals assessed by ELISA and PRNT (n=336). Column totals were used to calculate the percentages. ELISA IgG mean and standard deviation (SD) were calculated for each age group.

| **Age (years)** | **True Negative (n=39)** | **False Negative (n=24)** | **False Positive (n=28)** | **True Positive (n=245)** | **ELISA IgG (mean, SD)** | **Symptomatic dengue (n=20)** |
| --- | --- | --- | --- | --- | --- | --- |
| 2 (n=12) | 1 (2.6%) | 0 (0%) | 1 (3.6%) | 10 (4.1%) | 2.79 (1.00) | 0 (0%) |
| 3 (n=38) | 11 (28%) | 4 (17%) | 3 (11%) | 20 (8.2%) | 1.60 (1.16) | 2 (10%) |
| 4 (n=36) | 4 (10%) | 5 (21%) | 5 (18%) | 22 (9%) | 2.00 (1.28) | 1 (5%) |
| 5 (n=23) | 3 (7.7%) | 4 (17%) | 3 (11%) | 13 (5.3%) | 2.08 (1.40) | 2 (10%) |
| 6 (n=31) | 1 (2.6%) | 3 (12%) | 4 (14%) | 23 (9.4%) | 2.49 (1.06) | 1 (5%) |
| 7 (n=50) | 8 (21%) | 2 (8.3%) | 3 (11%) | 37 (15%) | 2.18 (1.25) | 3 (15%) |
| 8 (n=56) | 6 (15%) | 2 (8.3%) | 5 (18%) | 43 (18%) | 2.64 (1.18) | 3 (15%) |
| 9 (n=90) | 5 (13%) | 4 (17%) | 4 (14%) | 77 (31%) | 2.81 (1.07) | 8 (40%) |
