# Supplementary material for: Immunity to Non-Dengue Flaviviruses Impacts Dengue Virus Immunoglobulin G Enzyme-Linked Immunosorbent Assay Specificity in Cambodia
Source: J Infect Dis. 2024 Sep 19;231(2):e337–44. doi: 10.1093/infdis/jiae422 (PMC11841641; doi:10.1093/infdis/jiae422)
Supplement: jiae422_Supplementary_Data [file jiae422_supplementary_data.zip › TableS3.docx]

Supplemental Table 3. DENV antibody groups and presence of non-DENV neutralizing antibodies by age. Column totals were used to calculate the percentages.

| **Age (years)** | **DENV Naive (n=50)** | **False Positive (n=28)** | **Low DENV nAb**  **(n=21)** | **JEV nAb Positive (n=25)** | **ZIKV nAb Positive (n=9)** | **WNV nAb positive**  **(n=7)** | **≥ 1 Non-dengue Flavivirus Positive (n=32)** | **≥ 2 Non-dengue Flavivirus Positive**  **(N=7)** |
| --- | --- | --- | --- | --- | --- | --- | --- | --- |
| 2 (n=12) | 1 (2%) | 1 (3.6%) | 2 (9.5%) | 0 (0%) | 0 (0%) | 0 (0%) | 0 (0%) | 0 (0%) |
| 3 (n=38) | 7 (14%) | 3 (11%) | 4 (19%) | 2 (8%) | 0 (0%) | 1 (14%) | 2 (6.2%) | 1 (14%) |
| 4 (n=36) | 14 (28%) | 5 (18%) | 0 (0%) | 6 (24%) | 1 (11%) | 1 (14%) | 6 (19%) | 2 (29%) |
| 5 (n=23) | 7 (14%) | 3 (11%) | 3 (14%) | 5 (20%) | 2 (22%) | 1 (14%) | 7 (22%) | 1 (14%) |
| 6 (n=31) | 6 (12%) | 4 (14%) | 2 (9.5%) | 1 (4%) | 1 (11%) | 0 (0%) | 2 (6.2%) | 0 (0%) |
| 7 (n=50) | 8 (16%) | 3 (11%) | 3 (14%) | 3 (12%) | 0 (0%) | 0 (0%) | 3 (9.4%) | 0 (0%) |
| 8 (n=56) | 3 (6%) | 5 (18%) | 4 (19%) | 5 (20%) | 1 (11%) | 2 (29%) | 7 (22%) | 1 (14%) |
| 9 (n=90) | 4 (8%) | 4 (14%) | 3 (14%) | 3 (12%) | 4 (44%) | 2 (29%) | 5 (16%) | 2 (29%) |
